# Calcium/Cobalt Alginate Beads as Functional Scaffolds for Cartilage Tissue Engineering

**DOI:** 10.1155/2016/2030478

**Published:** 2016-01-06

**Authors:** Stefano Focaroli, Gabriella Teti, Viviana Salvatore, Isabella Orienti, Mirella Falconi

**Affiliations:** ^1^Department of Biomedical and Neuromotor Sciences, University of Bologna, 40126 Bologna, Italy; ^2^Department of Pharmacy and Biotechnology, University of Bologna, 40126 Bologna, Italy

## Abstract

Articular cartilage is a highly organized tissue with complex biomechanical properties. However, injuries to the cartilage usually lead to numerous health concerns and often culminate in disabling symptoms, due to the poor intrinsic capacity of this tissue for self-healing. Although various approaches are proposed for the regeneration of cartilage, its repair still represents an enormous challenge for orthopedic surgeons. The field of tissue engineering currently offers some of the most promising strategies for cartilage restoration, in which assorted biomaterials and cell-based therapies are combined to develop new therapeutic regimens for tissue replacement. The current study describes the* in vitro* behavior of human adipose-derived mesenchymal stem cells (hADSCs) encapsulated within calcium/cobalt (Ca/Co) alginate beads. These novel chondrogenesis-promoting scaffolds take advantage of the synergy between the alginate matrix and Co^+2^ ions, without employing costly growth factors (e.g., transforming growth factor betas (TGF-*β*s) or bone morphogenetic proteins (BMPs)) to direct hADSC differentiation into cartilage-producing chondrocytes.

## 1. Introduction

Articular cartilage covers the ends of bones in synovial joints and acts as a load-bearing material. Articular cartilage repair is one of the most challenging issues in the field of tissue regeneration because of the limited capacity of cartilage for self-regeneration once damaged [1–5]. Various surgical approaches are widely used to repair injured cartilage, including multiple drilling to encourage revascularization, abrasion arthroplasty, and perichondrial resurfacing. However, the efficacy of such strategies remains controversial, and these approaches are also unsatisfactory in terms of restoring the original structure and function of cartilage [6–10].

To overcome these drawbacks, cell-based therapy is currently under intense review for cartilage repair, and many different tactics and cell types have been explored for this purpose. Autologous chondrocyte implantation (ACI) was the first strategy employed in clinical practice, utilizing chondrocytes harvested from an area of the patient's own cartilage with diminished weight-bearing function [11–13]. However, several problems were reported with this technique, such as limited proliferative potential of the obtained chondrocytes and loss of functional cell phenotypes in culture [6, 11, 14].

Mesenchymal stem cells (MSCs) are a promising alternative cell source for cartilage repair. When appropriately stimulated, MSCs can differentiate into a variety of cell types, including cartilage-producing chondrocytes [4, 15–19]. MSCs are frequently isolated from bone marrow. Nevertheless, cell harvesting and isolation from bone marrow are associated with distinct disadvantages. For example, bone marrow aspiration can be painful for the patient, and the aspirates must be concentrated by using techniques that involve relatively high-cost instrumentation. Furthermore, MSC yields from bone marrow are quite low. Additional tissues have thus been proposed as a source of MSCs, including adipose tissue, which is abundant in adult stem cells, relatively easy to obtain from patients, and less expensive to handle than bone marrow for MSC isolation [4, 20–22]. In any case, common strategies to differentiate adult stem cells into chondrocytes still require the use of costly growth factors in the culture medium, such as transforming growth factor betas (TGF-*β*s), insulin-like growth factors, and bone morphogenetic proteins (BMPs). Of note, these additives could potentially lead to unexpected side effects during use in clinical practice [15, 23].

Recently, a number of research groups demonstrated that chondrogenic differentiation of MSCs can be achieved by maintaining the cells under hypoxic conditions [23–26], with the aim of reproducing the native environment of articular cartilage. Articular cartilage is an avascular tissue, deriving its oxygen supply from synovial fluid and subchondral bone. For this reason, the oxygen tension in the deepest layers of articular cartilage is no more than 1–6% [25, 27, 28]. Moreover, during endochondral bone formation, MSCs differentiate into chondrocytes that form a hyaline cartilage-rich matrix, which serves as a template for epiphyseal growth plate formation. These events occur during an avascular period in a decidedly hypoxic environment [22, 29].

The molecular mechanism associated with cell survival in the low oxygen environment involves the activation of the hypoxia inducible factor 1 (HIF-1) transcriptional complex. HIF-1 is a major mediator of the hypoxic response that is essential for chondrocyte differentiation and survival* in vivo*. HIF-1 contains two subunits: HIF-1*α* and HIF-1*β*. Under normoxic conditions, HIF-1*α* is rapidly degraded by prolyl-hydroxylase domain enzymes (PHDs) and factor inhibiting HIF (FIH) hydroxylase. The PHDs and FIH are inhibited at low oxygen tension; hence, HIF-1*α* escapes degradation and forms heterodimers with HIF-1*β*, permitting migration of the HIF-1*α*/HIF-1*β* complex into the nucleus and activation of target gene transcription, including that of cartilage-specific genes [22, 24, 26, 30].

Hypoxic conditions and HIF-1 upregulation can also be evoked by chemical induction; for instance, cobalt is well-known as a hypoxia-mimicking agent. This characteristic stems from the ability of cobalt ions (Co^+2^) to inactivate FIH by substitution for Fe^+2^ in the iron-binding center of the enzyme [31–33]. Regardless, the HIF-1-promoted differentiation of MSCs into chondrocytes is not sufficient for the ultimate purpose of restoring cartilage defects. Exogenously transplanted cells must also be supported by a biocompatible physical matrix (i.e., a scaffold), and selection of a suitable biomaterial for scaffolding is a critical factor in cartilage tissue engineering.

Alginate is widely used as a polymer for chondrogenic differentiation of MSCs [34–37]. This biomaterial is a naturally occurring heteropolysaccharide isolated from brown sea algae and is composed of *β*-D-mannuronic acid and *α*-L-guluronic residues. In the presence of divalent cations (e.g., calcium (Ca^+2^), barium (Ba^+2^), Co^+2^, and strontium (Sr^+2^)), alginate can be transformed into a hydrogel by ionic interactions between the *α*-L-guluronic residues of two distinct polymeric chains and the above-mentioned cations [35, 38–41]. Several tissue engineering studies have demonstrated that alginate provides an ideal environment to facilitate the spatial distribution of MSCs, resulting in a structural organization that resembles the native* in vivo* cartilage microenvironment [34–37]. Furthermore, alginate exerts chondroinductive actions to promote the synthesis of cartilage-specific matrix components [42–44].

The current study describes a new strategy to stimulate chondrogenic differentiation of commercially available human adipose-derived mesenchymal stem cells (hADSCs) via their encapsulation into a Ca/Co alginate bead scaffold. This approach takes advantage of the synergic effect of the alginate matrix and Co^+2^ ions on chondrogenesis and does not rely on TGF-*β*s, BMPs, or other exogenous growth factors or additives. Therefore, Ca/Co alginate bead scaffolds might be beneficial for prospective applications in articular cartilage repair.

## 2. Materials and Methods

### 2.1. hADSC Culture

hADSCs were purchased from a commercial source (Cat. number PT-5006; Lonza, Basel, Switzerland) and cultured in Dulbecco's modified Eagle's medium (DMEM; Gibco, Life Technologies, Monza, Italy) supplemented with 10% fetal bovine serum (FBS; Gibco, Life Technologies) and 1% penicillin/streptomycin in a humidified atmosphere (5% CO_2_) at 37°C. When the cells reached ~90% confluence, they were detached from the culture surface with 0.25% trypsin and subcultured. The hADSCs from passages 3–7 were used for further study.

### 2.2. Alginate Solution

Alginic acid sodium salt derived from brown algae (Na-alg) and suitable for cell encapsulation was obtained from Sigma-Aldrich (St. Louis, MO, USA). To prepare the Na-alg solution, 1% (w/v) of the solid sodium salt was dissolved in sterile 10 mM HEPES buffer (pH 7.4), and the mixture was filtered through a 0.2 mm membrane under sterile conditions.

### 2.3. Cell-Encapsulated Alginate Bead Production

hADSCs were detached from plastic tissue culture flasks by using 0.25% trypsin, resuspended at a density of 2 × 10^6^ cells/mL in sterile Na-alg solution, and dripped by 25-gauge needle into various gelling baths, all containing 200 mM CaCl_2_ and decreasing concentrations of CoCl_2_ (10, 5, 2.5, and 1.25 mM). The gelling baths were buffered with 10 mM HEPES. A 200 mM CaCl_2_ solution was used as the control gelling bath. The hADSC/Na-alg droplet suspension was maintained for 30 min at 37°C to form alginate beads. The resulting samples were designated Co10, Co5, Co2.5, Co1.25, and control based on the concentration of CoCl_2_ in the initial gelling bath. After washing the beads with HEPES buffer, DMEM supplemented with 10% FBS and 1% penicillin/streptomycin was added to tissue culture wells containing alginate-encapsulated cells. The prepared beads were incubated in a humidified environment (5% CO_2_) at 37°C. Cells within the beads were cultured for 7, 14, and 21 days, and the medium was changed every 3-4 days.

### 2.4. Morphology and Bead Size Distribution

Measurements of bead particle size (mean microsphere diameter) were performed by using a Nikon Eclipse E800 optical microscope (Nikon, Tokyo, Japan).

### 2.5. Assessment of Cell Viability and Cell Imaging

Cell viability was assessed up until day 21 of culture by using a LIVE/DEAD Viability/Cytotoxicity Assay kit (Life Technologies, Carlsbad, CA, USA). Encapsulated hADSCs were incubated for 15 min in HEPES buffer containing 0.1 *μ*M calcein AM, a cell-permeant fluorescent dye, and 0.1 *μ*M ethidium homodimer-1, a DNA-binding fluorescent dye. The samples were washed with HEPES buffer, transferred to a glass-bottomed 24-well plate, and immediately imaged by using a Nikon Eclipse E800 microscope. Live cells (green) and dead cells (red) were counted by using ImageJ software (National Institutes of Health, Bethesda, MD, USA). Cell viability was obtained by dividing the number of live cells by the total number of cells (live + dead).

### 2.6. Histological Analysis

Encapsulated cells were fixed at each experimental time point with 4% paraformaldehyde in 10 mM HEPES buffer for 2 h at 4°C. They were then dehydrated in an ascending series of alcohol solutions (50%, 70%, 90%, and 100%) and embedded in LR white resin (Sigma-Aldrich). Thin sections were prepared, stained with 1% toluidine blue, and observed by using a Nikon Eclipse E800 microscope.

### 2.7. Quantitative Real-Time Polymerase Chain Reaction (qRT-PCR)

To evaluate the differentiation capacity of the encapsulated hADSCs, samples were treated with 55 mM sodium citrate, 55 mM EDTA, and 0.9% NaCl in 10 mM HEPES-buffered saline (pH 6.8) with gentle shaking for 5 min. This resulted in formation of diluted Na-alg and the release of hADSCs from the alginate beads. At the end of each experimental time point, total RNA was extracted from the released cells by using a NucleoSpin RNA I kit (Macherey-Nagel, Duren, Germany). The RNA was then quantified by using a NanoDrop ND-1000 full-spectrum (ultraviolet/visible light) spectrophotometer (Thermo Scientific, Wilmington, DE, USA).

Next, cDNA was transcribed with reverse transcriptase SUPIII (Invitrogen, Carlsbad, CA, USA), and mRNA expression levels were analyzed via qRT-PCR by using a 7500 Real-Time PCR machine (Applied Biosystems, Life Technologies, Monza, Italy). The following TaqMan assays (Applied Biosystems, Life Technologies) were used for mRNA quantification: collagen type II (Col2A1; Hs00264051_m1), collagen type I (Col1A1; Hs00164004_m1), collagen type X (Col10A1; Hs00166657_m1), Sox9 (Sox9; Hs01001343_g1), versican (VCAN; Hs00171642_m1), and HIF-1*α* (HIF-1A; Hs00153153_m1). Relative gene expression levels were normalized to that of glyceraldehyde 3-phosphate dehydrogenase (GAPDH; Hs99999905_m1). Data are presented as fold changes relative to levels in control samples (cells encapsulated within alginate beads prepared with CaCl_2_ alone (Ca alginate beads) and cultured for the same amount of time) by using formula 2^−ΔΔCT^, as recommended by the manufacturer (User Bulletin number 2 P/N 4303859; Applied Biosystems).

### 2.8. Statistical Analysis

Statistical analysis of quantifiable data was conducted by performing an analysis of variance followed by Dunnett's multiple comparison test with GraphPad Prism 5.0 software (GraphPad Software, Inc., San Diego, CA, USA). Statistical differences between conditions were considered significant at *P* < 0.05.

## 3. Results

### 3.1. Size Distribution of hADSC-Loaded Capsules

**Figure 1:**
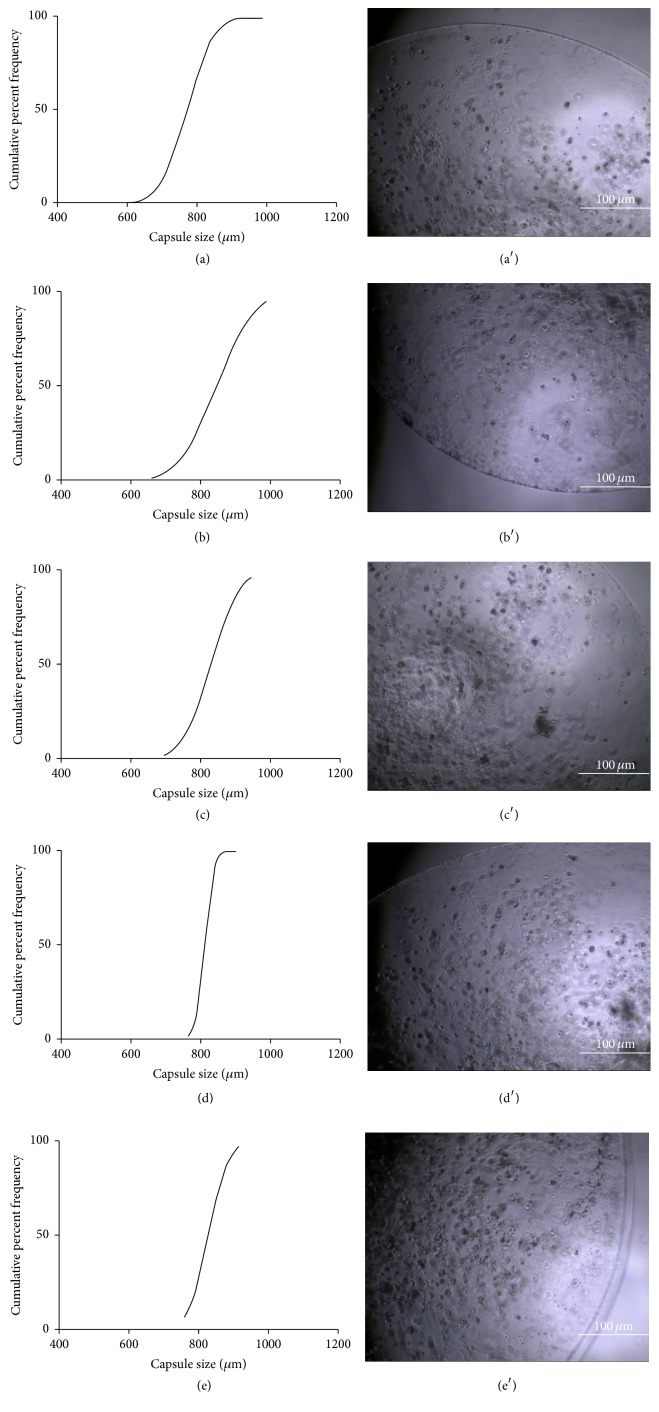
Cumulative size distribution (a–d) and microscopic images (a′–d′) of Ca/Co alginate beads with encapsulated hADSCs at 2 h after preparation. ((a), (a′)) Control sample; ((b), (b′)) Co1.25 sample; ((c), (c′)) Co2.5 sample; ((d), (d′)) Co5 sample; and ((e), (e′)) Co10 sample. The mean bead diameters were as follows: control, 810 ± 36 *μ*m; Co1.25, 821 ± 98; Co2.5, 789 ± 52; Co5, 826 ± 74 *μ*m; and Co10, 804 ± 16 *μ*m.

Alginate droplets containing encapsulated hADSCs were collected in gelling baths containing CaCl_2_ and varying amounts of CoCl_2_ so as to evaluate Co^+2^ concentration effects on the morphological characteristics of the particles. As a control, a gelling solution containing only CaCl_2_ was employed. First, the size distribution of the cell-encapsulated alginate beads was monitored, as shown in Figure 1. For all samples, the particle diameters were confined within a narrow size range, with a mean diameter ranging from 789 ± 52 to 826 ± 74 *μ*m. By comparison, the mean diameter of the control beads was 810 ± 36 *μ*m. These findings suggest that Co^+2^ concentration did not significantly affect capsule size.

**Figure 2:**
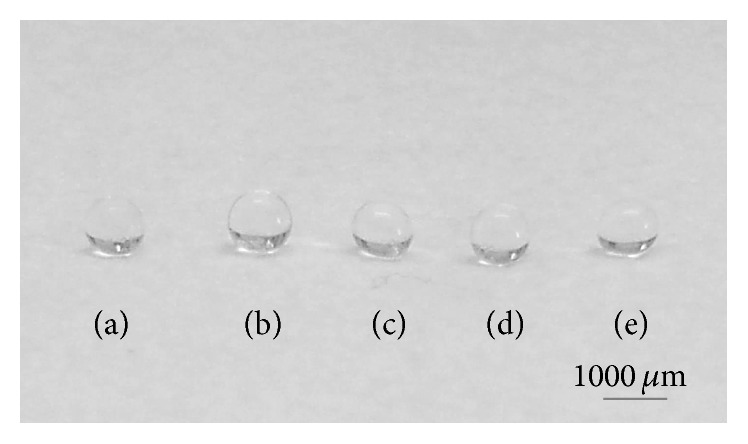
Images of Ca/Co alginate beads with encapsulated hADSCs on day 21 in culture. (a) Control sample; (b) Co1.25 sample; (c) Co2.5 sample; (d), Co5 sample; and (e) Co10 sample.

Light microscopic images showed a uniform cell distribution at 2 h after production of the hADSC-encapsulated alginate beads (Figures 1(a′)–1(e′)). Macroscopically (Figure 2), the particles maintained their spherical shape and exhibited a smooth surface. These proprieties were maintained during the 21 days in culture, and no macroscopic evidence of particle degradation or deformation was noted.

### 3.2. Cell Viability

**Figure 3:**
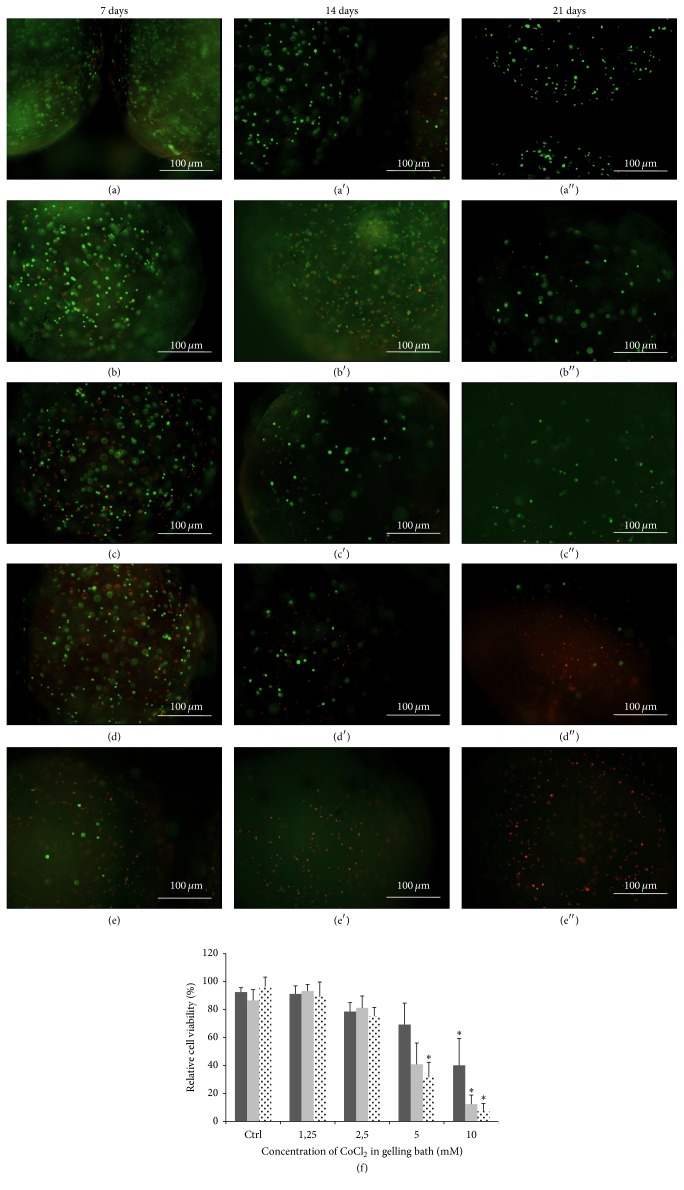
Live/dead (green/red) staining of hADSCs encapsulated within alginate Ca/Co beads. ((a), (a′), (a′′)) Control sample; ((b), (b′), (b′′)) Co1.25 sample; ((c), (c′), (c′′)) Co2.5 sample; ((d), (d′), (d′′)) Co5 sample; and ((e), (e′) (e′′)) Co10 sample. (f) Cell viability of hADSCs (live cells/live + dead cells) encapsulated within Ca/Co alginate beads. Black bar: 7 days; dark grey bar: 14 days; light grey bar: 21 days. Data are given as the means ± the standard deviation (SD) (*n* = 3 independent experiments); *∗* indicates statistical differences compared to control samples at the same time point (*P* < 0.05).

To evaluate the potential toxicity of Co^+2^ ions against encapsulated hADSCs, a calcein AM/ethidium homodimer-1 assay was performed. Images of the stained cells within the alginate beads are shown in Figure 3, where live cells are green and dead cells are red. The images were employed to measure cell viability at 7, 14, and 21 days in culture (Figure 3(f)). The live/dead cell ratios for hADSCs encapsulated within the Co1.25 and Co2.5 beads did not differ significantly from that in the corresponding controls, although the ratio appeared to be slightly less than the control ratio in the Co2.5 group at all the time points examined. Cell viability in the Co5 sample was comparable to that in the control at 7 days in culture, but the number of live cells decreased at 14 and 21 days, with a significant difference from the control at 21 days. Cells in the Co10 sample showed a relative cell viability of 40.13%, 12.36%, and 7.24% on days 7, 14, and 21, respectively, which was significantly lower than the control at each time point, indicating the potential toxicity of Co^+2^ contained within the alginate beads (Figure 3).

### 3.3. Histological Observations

**Figure 4:**
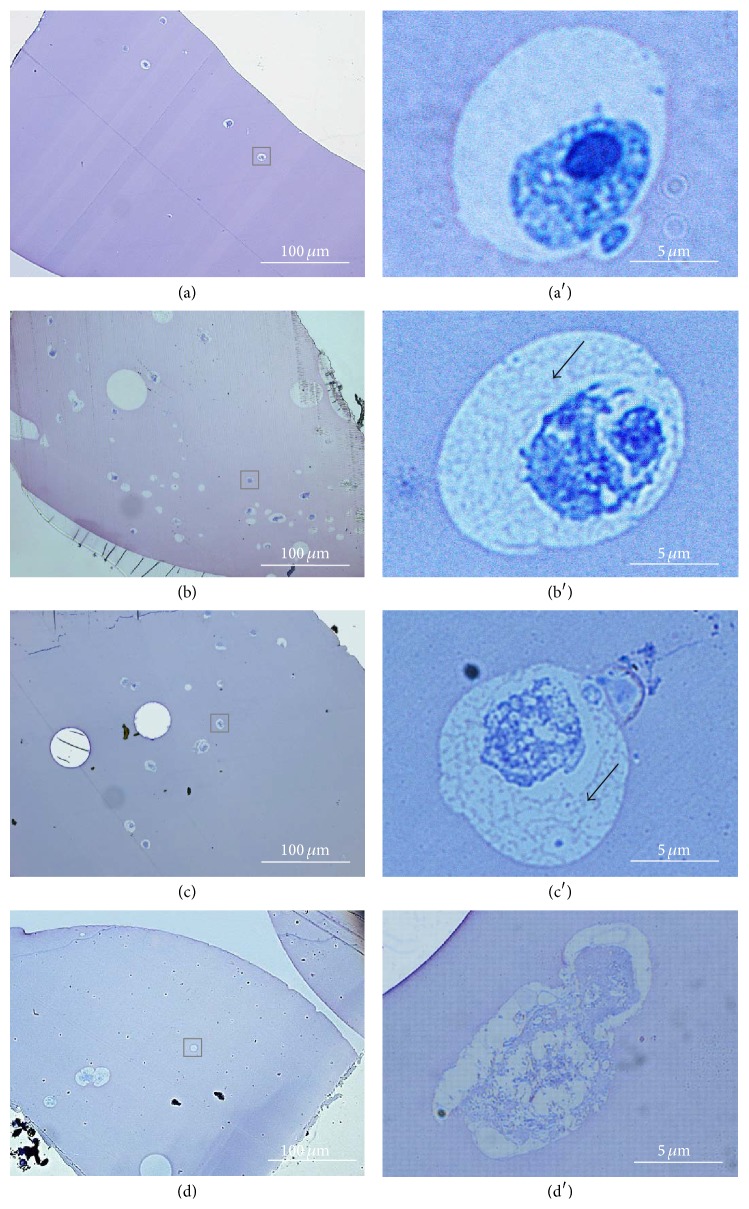
Microscopic images of hADSCs encapsulated within Ca/Co alginate beads at 21 days in culture and stained with toluidine blue. ((a), (a′)) Co1.25 sample; ((b), (b′)) Co2.5 sample; ((c), (c′)) Co5 sample; and ((d), (d′)) Co10 sample. Black arrows indicate proteoglycan matrix deposition. Black squares indicate lacunae.

The round shape of the hADSCs within the alginate beads and the unfilled spaces, or lacunae, associated with cartilage tissue-like structures were both illustrated by toluidine blue staining (Figure 4). After 21 days in culture, lacunae and matrix deposition were clearly revealed in the Co2.5 sample. The Co5 beads demonstrated similar cell behavior as the Co2.5 beads, even though the number of lacunae in the encapsulated cells was lower than that observed for the Co2.5 sample. The numbers of encapsulated cells within the Co1.25 and Co10 beads were quite limited, and the lacunae had an irregular appearance in the Co10 sample and were not well defined.

### 3.4. qRT-PCR Analysis of mRNA Expression Levels of Chondrogenic Markers in hADSCs

**Figure 5:**
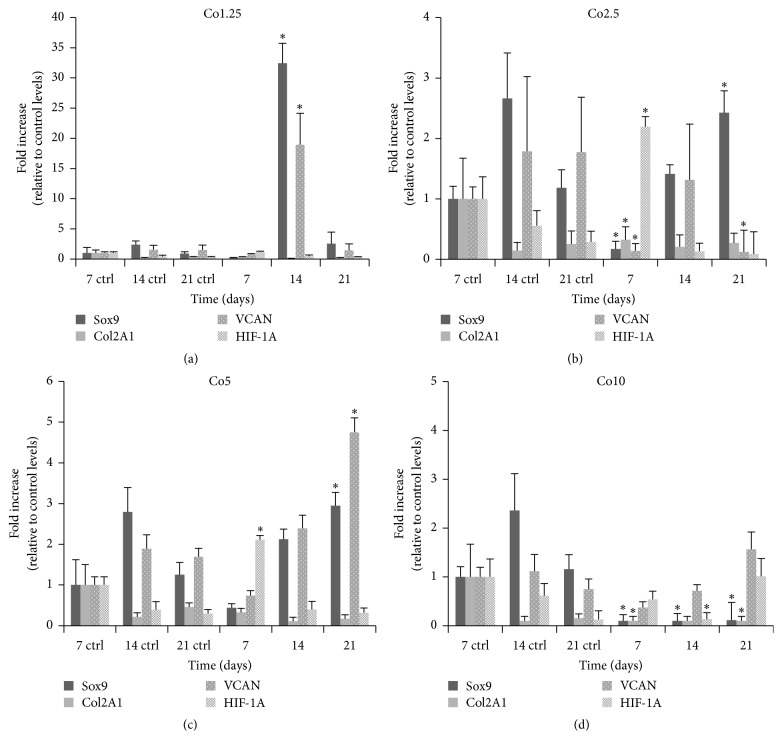
qRT-PCR analysis of hADSCs encapsulated within Ca/Co alginate beads. Expression levels of marker genes were normalized to that of GAPDH and calculated as fold changes relative to expression levels of hADSCs encapsulated within control Ca alginate beads at 7 days. Data are given as means ± the SD (*n* = 3 independent experiments; ^*∗*^ *P* < 0.05). VCAN: versican.

**Figure 6:**
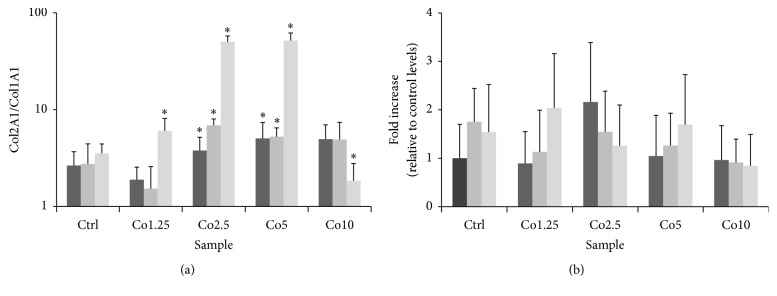
(a) Col1A1/Col1A2 mRNA ratio synthetized by hADSCs encapsulated within Ca/Co alginate beads. (b) qRT-PCR analysis of Col10A1. Expression levels of marker genes were normalized to that of GAPDH and calculated as fold changes relative to expression levels of hADSCs encapsulated within control Ca alginate beads at 7 days. Black bar: 7 days; dark grey bar: 14 days; light grey bar: 21 days. Data are given as means ± the SD (*n* = 3 independent experiments; ^*∗*^ *P* < 0.05).

To verify hADSC differentiation into chondrocytes, mRNA expression levels of HIF-1, chondrogenic markers (collagen type II, Sox9, and versican), and chondrogenic hypertrophic marker (collagen type 10) were quantified in the cells by qRT-PCR. The Sox9 mRNA expression profile in the control samples showed a peak at 14 days in culture, followed by downregulation at 21 days. Gene expression of collagen type II and HIF-1 decreased at 14 and 21 days, while that of versican remained unaltered over the 21-day culture period (Figure 5). The Co1.25 sample exhibited strong upregulation of Sox9 and versican gene expression at 14 days, yielding mRNA levels that were 30- and 18-fold higher than control levels, respectively. However, Sox9 and versican mRNA expression levels were similar to control levels at day 21, and HIF-1 and collagen type II gene expression did not vary significantly over the course of the experiment (Figure 5(a)). Sox9 gene expression in the Co2.5 sample continuously increased from day 7 to day 21, while the control expression levels decreased from day 14 to day 21. HIF-1 mRNA levels showed upregulation at 7 days, but the expression levels decreased at 14 and 21 days, and were similar to control levels (Figure 5(b)). Sox9 and versican mRNA levels both showed continuous increases in the Co5 sample (Figure 5(c)), and versican mRNA levels showed the same trend in the Co10 sample (Figure 5(d)). In the Co5 sample (Figure 5(c)), versican mRNA expression levels were ~3-fold higher than the control level at 21 days in culture. Meanwhile, HIF-1 showed a similar trend as in the Co2.5 sample, with a ~2-fold increase at 7 days in culture, followed by downregulation on days 14 and 21. The Col2A1/Col1A1 ratio showed no significant differences in Co1.25 at 7 and 14 days of culture with respect to control and it increased after 21 days of culture; differently the ratio in Co2.5 and Co5 resulted in being higher than control in all experimental times whereas the Co10 values were similar to control, except for day 21 where a decrease was shown (Figure 6(a)). Concerning the Col10A1, its expression resulted similar in all experimental conditions (Figure 6(b)).

## 4. Discussion

The main goal of the present work was to induce chondrogenic differentiation of hADSCs by employing low-cost alginate materials and straightforward techniques. Alginate hydrogels are widely used as scaffolds in tissue engineering applications because they provide a three-dimensional structure reminiscent of the native extracellular matrix of cells within tissues. Alginate also has the ability to promote and stabilize the chondrogenic phenotype [14, 35, 43, 44]. Here, we exploited the synergic effect of alginate in combination with Co^+2^ ions to mimic the natural environment and biophysical properties of cartilage tissue* in vivo*. Due to its negative charge and abundance of hydroxyl functional groups, alginate shows a high affinity toward bivalent ions, which then trigger gel formation by generation of interchain bridges after contact with the polysaccharide [35, 38–41].

Divalent ions not only are adsorbed onto the surface of the biomaterial in contact with the gelling bath solution, but also diffuse into the gel through microscopic channels. In this manner, the ions can interact with functional groups inside the gel [45]. This process permits the formation of a uniform gel structure, where the ions are homogeneously distributed. In this study, the alginate gel was obtained by using CaCl_2_ and varying concentrations of CoCl_2_. Co^+2^ ions, like Ca^+2^ ions, participate in the gelation process by producing an* in situ* Co^+2^ reservoir directly available to the encapsulated cells.

Keeping our low-cost philosophy in mind, we produced cell-encapsulated alginate beads via a dripping technique. This method is widely used in cell encapsulation because it is easy to set up and requires no expensive instrumentation [46, 47]. Although some researchers prefer other approaches to generate alginate particles (e.g., emulsification or electrostatic droplet generation techniques [48–50]), our experimental conditions yielded alginate beads with a narrow size distribution and a smooth spherical shape, two critical parameters for quality control of three-dimensional cell culture scaffolds [46, 51, 52] (Figures 1 and 2). Therefore, the dripping method is reproducible and suitable for our aims.

The ideal conditions for survival of encapsulated cells were empirically determined by assessing relative cell viability within alginate beads collected in gelling baths containing varying amounts of Co^+2^. Consequently, the live/dead cell assay showed a dose-response relationship with Co^+2^ concentration, with enhanced viability of hADSCs in Co1.25 and Co2.5 alginate spheres after 21 days in culture. These findings suggest that initial Co^+2^ concentrations of 1.25 and 2.5 mM in the gelling bath are well tolerated by the cells. However, cell viability was reduced in the Co5 and Co10 samples, indicating that high Co^+2^ concentrations are not ideal for long-term study of chondrogenic differentiation. For example, the live/dead cell ratio in the Co10 sample was ~45% at 7 days and decreased thereafter, ascribable to an acute cytotoxic effect of Co^+2^ at elevated concentrations.

Importantly, an equilibrium exists between the concentration of Co^+2^ in the gelling bath and that inside the alginate bead. We assumed that the Co^+2^ concentration within the particle would be less than that in the gelling bath, allowing cell survival and differentiation. Regardless, future studies will be required to investigate the optimal Co^+2^ concentration within the beads for chondrocytic differentiation of encapsulated stem cells.

Chondrogenic differentiation was monitored herein by qRT-PCR. HIF-1 gene expression was of particular interest because HIF-1 is a predominant mediator of the hypoxic response. As noted above, the HIF-1*α* subunit is rapidly degraded under normoxic conditions by PHDs and FIH. Under hypoxic conditions, PHDs and FIH are inactivated and HIF-1*α* is spared; hence, HIF-1*α* can interact with HIF-1*β* and translocate into the nucleus. The HIF-1*α*/HIF-1*β* complex then binds to hypoxia-responsive elements in cartilage marker genes, enhancing their transcription [19, 22, 26, 30].

Intriguingly, Co^+2^ ions can reportedly increase HIF-1 mRNA synthesis [31]. In our experiments, HIF-1 mRNA expression levels were increased at 7 days in culture in the Co2.5 and Co5 samples and decreased thereafter. These results are suggestive of a negative HIF-1 feedback mechanism with prolonged hypoxic exposure. On the other hand, Sox9 mRNA expression levels were continuously upregulated in the Co2.5 and Co5 samples over the 21 days of the experiment, while the same trend was observed for versican mRNA levels in the Co5 sample. These two markers have pivotal functions during the early stages of chondrogenic differentiation: Sox9 is a transcription factor that regulates cell condensation and the production of other chondrogenic markers, such as collagen type II [53, 54], while versican is a hyaluronan-binding proteoglycan that plays specific roles at the articular cartilage surface and is involved in regulation of the cartilage cell phenotype [54–56].

Although the Co1.25 sample showed strong upregulation of Sox9 mRNA and versican mRNA at 14 days, no significant change in HIF-1 mRNA expression was observed at any time point. We surmise that the Co^+2^ concentration in the Co1.25 capsule was insufficient to promote chondrogenic differentiation and that the increased gene expression of chondrogenic markers in hADSCs resulted from induction by alginate alone.

Toluidine blue staining and histological observations confirmed the impact of the Co2.5 and Co5 samples on the early stages of chondrogenic differentiation. Both samples promoted the formation of numerous lacunae associated with cartilage tissue-like structures at day 21 in culture, as well as proteoglycan matrix deposition (Figures 4(b), 4(b′) and 4(c), 4(c′)). However, in agreement with the qRT-PCR results, no proteoglycan deposition into the lacunae was observed for the Co1.25 sample. Moreover, the irregular morphology of the cells in the Co10 sample again suggests that the conditions in this scaffold were inappropriate for optimal hADSC differentiation.

Collagen type II mRNA was only detected at low levels in all of the samples, even though collagen type II is a fundamental component of the cartilage extracellular matrix. Possibly, optimal collagen type II synthesis requires more time than the 21-day observation period of our study, depending on the culture system [37]. However, the Col1A1/Col2A1 ratio suggests that Co2.5 and Co5 samples may be more suitable for chondrogenic differentiation; in fact, a ~50-fold increase of Col2A1 in comparison with Col1A1 after 21 days could reveal a tendency of hADSCs to evolve in chondrocytes. Nevertheless, further investigations will be focused on the long-term maintenance of hADSCs within Co/Ca alginate beads to evaluate Col2A1 synthesis.

Finally, the expression of collagen type X was detected. This protein is expressed when chondrocytes hypertrophy. Generally, collagen type X is restricted to the deep cartilage zone and the adjacent calcified cartilage in adult articular cartilage. A hypertrophic chondrocyte is a cell that has gradually differentiated toward osteogenesis [57, 58]. In our experiments, the collagen type 10 expression is very low and similar to the control in all experimental conditions. The results reveal the beads have the advantage of keeping the differentiated cells in a chondrocyte or chondroprogenitor phenotypes without differentiating toward osteogenesis. However, as stated above, long-term studies are necessary.

## 5. Conclusions

This study shows a novel and low-cost approach to induce* in vitro* chondrogenic differentiation of MSCs encapsulated within alginate beads. This strategy exploits the synergic actions of Co^+2^ and alginate and does not include traditional differentiation-promoting growth factors. The alginate beads produced herein provide a cartilage tissue-mimetic environment and are promising for use in cartilage tissue engineering applications. In addition, the dripping technique used for bead production is straightforward, reproducible and permits gentle encapsulation of hADSCs without reducing cell viability. However, this work only represents an initial phase of our study of alginate beads for cartilage tissue engineering; the results suggest a chondroprogenitor phenotype of cells and the complete differentiation requires long-term experiments. Optimization of the Co^+2^ molar concentration in the beads and long-duration culturing are already underway in a continuation of the current investigation.
